# Phenotypic and molecular characterization of Hessian fly resistance in diploid wheat, *Aegilops tauschii*

**DOI:** 10.1186/s12870-019-2058-6

**Published:** 2019-10-22

**Authors:** Jill A. Nemacheck, Brandon J. Schemerhorn, Steven R. Scofield, Subhashree Subramanyam

**Affiliations:** 1 USDA-ARS Crop Production and Pest Control Research Unit, West Lafayette, IN 47907 USA; 2 Department of Entomology, Purdue University, West Lafayette, IN 47907 USA; 3 Department of Agronomy, Purdue University, West Lafayette, IN 47907 USA

**Keywords:** Insect resistance, Biotic stress, qRT-PCR, Surrogate, Functional genomics, Permeability, Oxidative stress, Secondary metabolites, Lectins

## Abstract

**Background:**

The Hessian fly (*Mayetiola destructor*), belonging to the gall midge family (Cecidomyiidae), is a devastating pest of wheat (*Triticum aestivum*) causing significant yield losses. Despite identification and characterization of numerous Hessian fly-responsive genes and associated biological pathways involved in wheat defense against this dipteran pest, their functional validation has been challenging. This is largely attributed to the large genome, polyploidy, repetitive DNA, and limited genetic resources in hexaploid wheat. The diploid progenitor *Aegilops tauschii*, D-genome donor of modern-day hexaploid wheat, offers an ideal surrogate eliminating the need to target all three homeologous chromosomes (A, B and D) individually, and thereby making the functional validation of candidate Hessian fly-responsive genes plausible. Furthermore, the well-annotated sequence of *Ae. tauschii* genome and availability of genetic resources amenable to manipulations makes the functional assays less tedious and time-consuming. However, prior to utilization of this diploid genome for downstream studies, it is imperative to characterize its physical and molecular responses to Hessian fly.

**Results:**

In this study we screened five *Ae. tauschii* accessions for their response to the Hessian fly biotypes L and *vH13*. Two lines were identified that exhibited a homozygous resistance response to feeding by both Hessian fly biotypes. Studies using physical measurements and neutral red staining showed that the resistant *Ae. tauschii* accessions resembled hexaploid wheat in their phenotypic responses to Hessian fly, that included similarities in larval developmental stages, leaf and plant growth, and cell wall permeability. Furthermore, molecular responses, characterized by gene expression profiling using quantitative real-time PCR, in select resistant *Ae. tauschii* lines also revealed similarities with resistant hexaploid wheat.

**Conclusions:**

Phenotypic and molecular characterization of *Ae. tauschii* to Hessian fly infestation revealed resistant accessions that shared similarities to hexaploid wheat. Resembling the resistant hexaploid wheat, the *Ae. tauschii* accessions mount an early defense strategy involving defense proteins including lectins, secondary metabolites and reactive oxygen species (ROS) radicals. Our results reveal the suitability of the diploid progenitor for use as an ideal tool for functional genomics research in deciphering the wheat-Hessian fly molecular interactions.

## Background

The Hessian fly, *Mayetiola destructor* (Say), belonging to the gall midge family Cecidomyiidae (order: Diptera), is a destructive pest of hexaploid bread wheat (*Triticum aestivum* L.) in the United States and other parts of the world [1, 2], causing significant economic damage [3]. Being an obligate parasite, the Hessian fly receives all of its nutrition from the plant. The adult females lay eggs primarily on the adaxial surface of the leaves where they hatch. The newly hatched 1st-instar larvae (neonates) crawl towards the base of the plant, where they establish sustained feeding sites. Probing of the host plant by the Hessian fly larvae yields either an incompatible (avirulent larvae; resistant wheat) or compatible (virulent larvae; susceptible wheat) interaction. On resistant wheat, the larvae die within 4–5 days after egg hatch (DAH) appearing as dead, red larvae; however, on susceptible wheat the larvae go through two more instars before they pupate to adults, thus completing their development (see review, [4]).

The wheat-Hessian fly interaction fits the gene-for-gene model with the recognition of the larval avirulence gene product by the host-resistance product [5]. The most effective, and economical way to manage this insect pest is by deploying resistant wheat cultivars harboring Hessian fly resistance (*H*) genes [2, 6], with 35 genes (*H1* to *H34* plus *Hdic*) being documented so far [7–9]. However, deployment of resistant cultivars with high level of antibiosis to the larvae exerts strong selection pressure on Hessian fly populations, favoring the selection of virulent biotypes [10] that can overcome deployed resistance, posing a threat to long-term production of wheat.

An alternate strategy to enhance and complement native or introgressed *H* gene resistance is by employing forward genetics to develop wheat lines overexpressing candidate defense-response genes or negatively regulating genes involved in wheat susceptibility to Hessian fly. Despite characterization of several candidate Hessian fly-responsive genes in hexaploid wheat cultivars, their functional validation through supplementation and/or mutational approaches are challenging due to: (i) large genome size (~ 17 Gb), (ii) allohexaploid genome (AABBDD), (iii) 85% repetitive DNA, and (iv) limited availability of genetic and genomic resources [11, 12]. We recently proposed the suitability of *Brachypodium distachyon* as an alternate surrogate for undertaking functional analysis of Hessian fly-responsive genes [13]. However, unlike wheat, *B. distachyon* is a nonhost exhibiting molecular responses intermediate to resistance and susceptibility [13, 14], therefore making the functional genomics of Hessian fly-responsive genes limited in scope. Another approach would be the utilization of diploid wheat *Aegilops tauschii* (goat grass) genome, which shares a close relationship with hexaploid wheat, for cloning and manipulating candidate Hessian fly-responsive genes via modern biotechnological tools, as an alternative model system for bread wheat.

*Ae. tauschii* Coss. (2n = 2x = 14, genome DD) is the diploid progenitor of the D-genome donor of modern-day hexaploid bread wheat (*T. aestivum*, 2n = 6x = 42, genome AABBDD). It is an important genetic resource for wheat and harbors useful genes against several biotic stressors [15–18]. In fact, several of the Hessian fly resistance genes including *H13, H22, H23, H24, H26*, and *H32* have been introgressed into hexaploid wheat from *Ae. tauschii* [19]. Furthermore, several of the Hessian fly-responsive defense genes are mapped to the D-genome [20, 21]. The recent sequencing of *Ae. tauschii*, provides insight into the structure and organization of this diploid genome [22]. Additionally, a Till-D (Targeting Induced Local Lesions in Genomes, TILLING) population for *Ae. tauschii* has been developed recently [23] that offers a powerful genetic approach for functional analysis of wheat genes.

A first step towards utilization of this diploid genome for further genomics research in wheat-Hessian interactions requires evaluation and identification of Hessian fly resistant and susceptible *Ae. tauschii* accessions and deciphering their response to larval feeding. In the current study we have characterized the phenotypic and molecular responses of five *Ae. tauschii* accessions to two Hessian fly stocks, field-collected biotype L, which is the most virulent Hessian fly biotype [24], and lab-cultured *vH13* stock. A previous study documented the responses of several *Ae. tauschii* accessions to Hessian fly larval feeding [15], using biotype D, to identify new genetic sources of resistance that could be potentially transferred to synthetic hexaploid wheat for developing Hessian fly-resistant cultivars. However, unlike our study, this work did not attempt to dissect molecular pathways associated with the resistance. We undertook transcript profiling studies for genes that serve as biomarkers for compatible and incompatible interactions in hexaploid wheat, as well as genes involved in key defense responses during biotic stress, including secondary metabolites and oxidative stress. Our results identified two and four *Ae. tauschii* accessions that were homozygous resistant to *vH13* and biotype L Hessian fly stocks, respectively. Further, transcript profiling studies of Hessian fly-responsive genes in these resistant *Ae. tauschii* accessions revealed similarities to expression patterns observed in hexaploid *T. aestivum* wheat, thereby suggesting the suitability of this diploid genome as an alternate model for functional genomics research in deciphering the wheat-Hessian fly molecular interactions.

## Results

### Phenotypic response of *Ae. tauschii* to Hessian fly larval feeding

#### Reaction to Hessian fly infestation

**Fig. 1:**
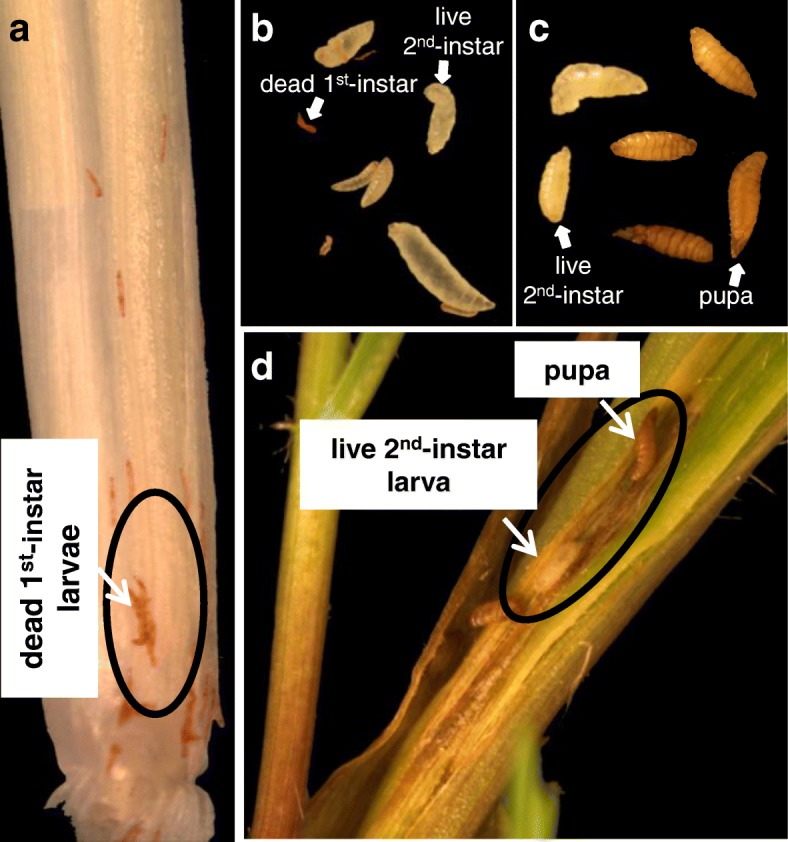
Phenotypic response of *Ae. tauschii* to Hessian fly larval feeding. *Ae. tauschii* accessions showed homozygous resistance response or mixed response to feeding by biotype L and *vH13* Hessian fly larvae. **a** Representative resistance response plant having only dead 1st-instar larvae at the base of the crown tissue (the larval feeding site); **b** Mix of dead, red 1st-instar larvae and white 2nd-instar larvae removed from a representative heterozygous plant (TA2452) at 7 DAH; **c** Mix of 2nd-instar white larvae and pupae removed from a representative heterozygous plant (TA2452); **d** Representative mixed response (TA2452) biotype L-infested plant showing presence of white 2nd-instar larva and pupae by 17 DAH

Five *Ae. tauschii* accessions, TA2452 (*H13*), TA1644 (*H22*), TA2473 (*H26*), TA1651 (*H32*), and TA1642 (*H23*), that are donors to known Hessian fly resistance genes, were selected to evaluate their reaction to infestation by two biotypes, L and *vH13* (Table 1). Plants from the accessions TA2473 and TA1651 were homozygous resistant (where all larvae die in the 1st-instar developmental stage) to both Hessian fly biotypes used in the current study (Table 1). By 7 DAH larvae on all plants were avirulent, appearing as dead, red larvae (Fig. 1a). By 17 DAH, these larvae had rapidly shriveled, decomposed and disappeared. However, plants of TA2452 exhibited a mixed response comprising of resistant plants (homozygous), as well as plants having dead (avirulent, red) and live 2nd-instar (virulent, white) larvae on the same leaf sheath (classified as heterozygous), by 7 DAH following infestation with both biotype L and *vH13* flies (Table 1). At 7 DAH, 40% the TA2452 plants were homozygous resistant (having only avirulent larvae) and 60% plants were heterozygous as they harbored both dead and virulent 2nd-instar larvae on the same leaf sheath (Fig. 1b) in response to biotype L infestation (Table 1). In response to *vH13* flies, 86.7% of TA2452 plants were heterozygous with live and dead larvae and only 13.3% plants were homozygous resistant (Table 1). The live 2nd-instar virulent biotype L and *vH13* larvae were present on the heterozygous plants even at 17 DAH. Around 11.6% of the biotype L larvae successfully pupated, while *vH13* larvae were still in the 2nd-instar stage, by 17 DAH. By 24 DAH around 6.9% of *vH13* larvae pupated (Fig. 1c, d). Plants for TA1644 and TA1642 were also homozygous resistant in response to biotype L attack with all larvae dying by 7 DAH. However, these accessions showed a mixed response to feeding by *vH13* (Table 1). At 7 DAH, 86.7 and 37.5% of TA1644 and TA1642 plants, respectively, were homozygous resistant and 13.3% of TA1644 and 62.5% of TA1642 plants were heterozygous with both live and dead larvae on the same plant (Table 1). Similar to TA2452, several of the *vH13* larvae also survived on TA1644 (20.5%) and TA1642 (11.1%) plants till 24 DAH. While the surviving *vH13* 2nd-instar larvae on TA1642 plants pupated, the 2nd-instar larvae on TA1644 plants failed to pupate.

**Table 1:** Phenotypic response of *Ae. tauschii* wheat accessions to Hessian fly larval feeding

Accession No.	*H* gene^a^/chromosome	No. of plants evaluated	No. of dead:live larvae	Mean no. larvae/ plant	No. of homo:het^b^plants	Necrotic lesions
a) Response to biotype L						
TA2452	*H13*/6D	15	302:73	25	6:9	P (40%)
**TA1644**	***H22*** **/1D**	**15**	**367:0**	**24**	**15:0**	**A**
**TA2473**	***H26*** **/3D**	**15**	**284:0**	**19**	**15:0**	**A**
**TA1651**	***H32*** **/3D**	**15**	**218:0**	**15**	**15:0**	**A**
**TA1642**	***H23*** **/6D**	**15**	**210:0**	**14**	**15:0**	**A**
b) Response to biotype *vH13*						
TA2452	*H13*/6D	15	162:119	19	2:13	P (27%)
TA1644	*H22*/1D	15	205:20	15	13:2	P (33%)
**TA2473**	***H26*** **/3D**	**10**	**174:0**	**17**	**10:0**	**A**
**TA1651**	***H32*** **/3D**	**15**	**186:0**	**12**	**15:0**	**A**
TA1642	*H23*/6D	8	97:31	16	3:5	P (38%)

*homo* homozygous resistant, *het* heterozygous, *P* present, *A* absent

^a^donor for Hessian fly resistance (*H*) gene and chromosome mapped to

^b^plants were counted as het if they had both dead and live larvae

Numbers in parentheses represent percent plants showing presence of necrotic lesions on leaf surface

Bold font indicates 100% homozygous resistant lines

**Fig. 2:**
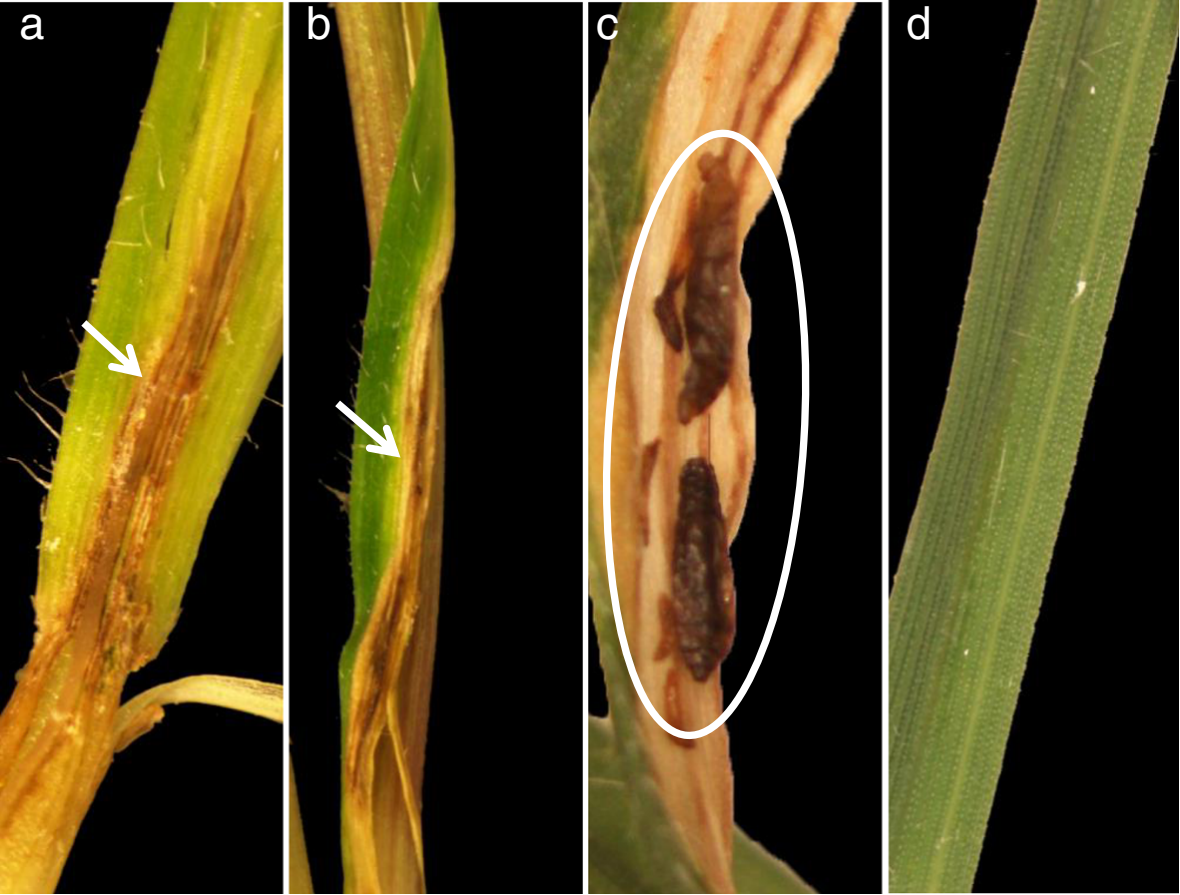
Necrotic lesions on Hessian fly infested *Ae. tauschii* accessions. Representative TA2452 heterozygous plants showing presence of lesions, visible as dark necrotic patches, in response to feeding by **a**) biotype L and **b**) *vH13* Hessian fly larvae. **c**) Larvae and pupae inhabiting the sites of necrotic lesions. **d**) Representative TA2473 resistant plant lacking development of necrotic lesions in response to Hessian fly larval feeding

The five *Ae. tauschii* accessions were evaluated for their ability to produce lesions as an indication of hypersensitive response (HR) to Hessian fly larval attack. Visible lesions (dark necrotic patches) were observed only in accessions that showed a mixed response to Hessian fly infestation (Table 1). In the accessions exhibiting a mixed response, lesions were present mostly on heterozygous plants having both live and dead larvae, while very few of the resistant plants (all larvae dead) showed necrotic lesions. In TA2452, 40 and 27% of plants showed lesions in response to biotype L (Fig. 2a) and *vH13* larval feeding (Fig. 2b), respectively. Such necrotic lesions were also observed in lines TA1644 (33%) and TA1651 (38%) showing mixed responses to feeding by *vH13* larvae (Table 1). Furthermore, several of the live larvae and pupae were also observed at the sites of these necrotic patches (Fig. 2c). Interestingly, none of the homozygous resistant *Ae. tauschii* accessions exhibited similar necrotic lesions on the leaf sheath (Table 1, Fig. 2d).

#### Leaf and plant growth

**Fig. 3:**
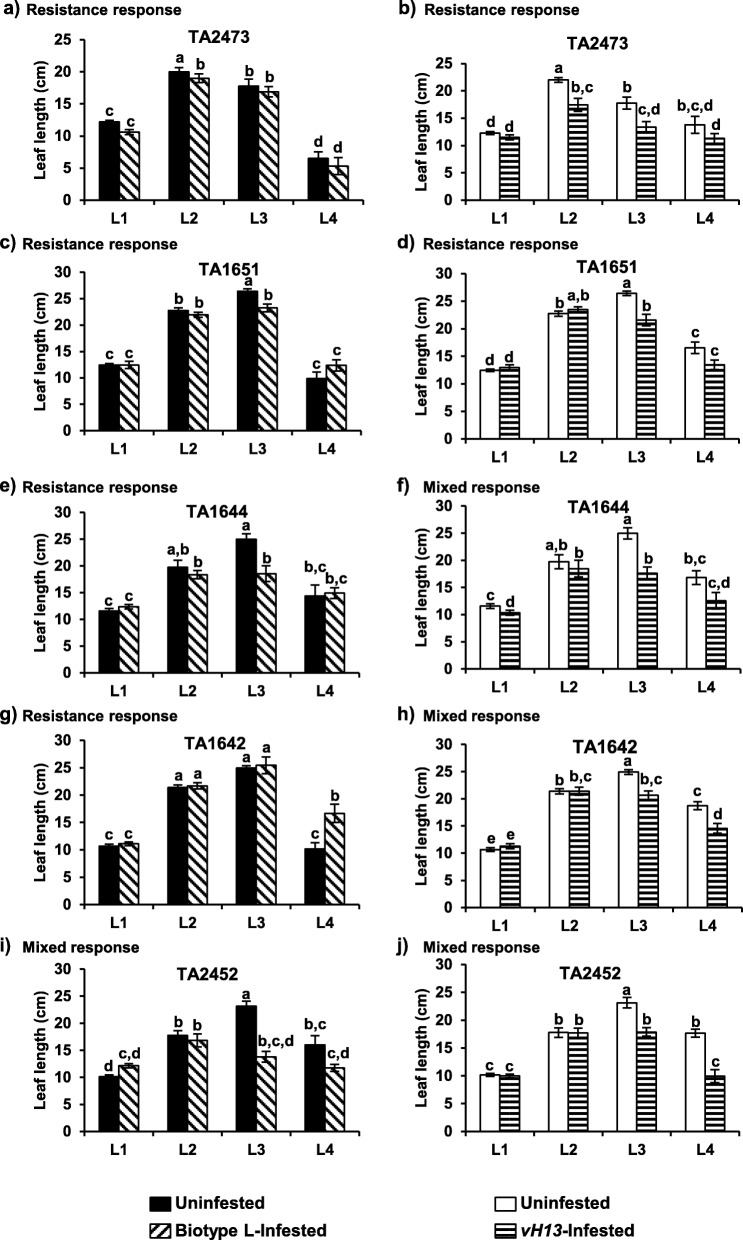
Leaf growth in Hessian fly infested *Ae. tauschii* accessions. Plants from *Ae. tauschii* lines TA2473 (**a**, **b**), TA1651 (**c**, **d**), TA1644 (**e**, **f**), TA1642 (**g**, **h**), and TA2452 (**i**, **j**) were infested with biotype L (left panel) and *vH13* (right panel) Hessian fly stocks. Nondestructive leaf (L1: leaf 1; L2: leaf 2; L3: leaf 3; L4: leaf4) measurements from soil level to leaf blade tips were taken at 17 and 24 DAH, for biotype L- and *vH13*-infested plants, respectively. Measurements were also taken from uninfested control plants similarly for the same time-points. Data are represented as mean ± standard error (SE). The letters at the top of the bars indicate significant differences based on Tukey’s HSD test (*p* < 0.05). Same letters indicate no difference between the two treatments. Different letters indicate significant differences between the two treatments. Black and white bars indicate uninfested control plants. Diagonal and parallel bars indicate biotype L- and *vH13*-infested plants, respectively

**Fig. 4:**
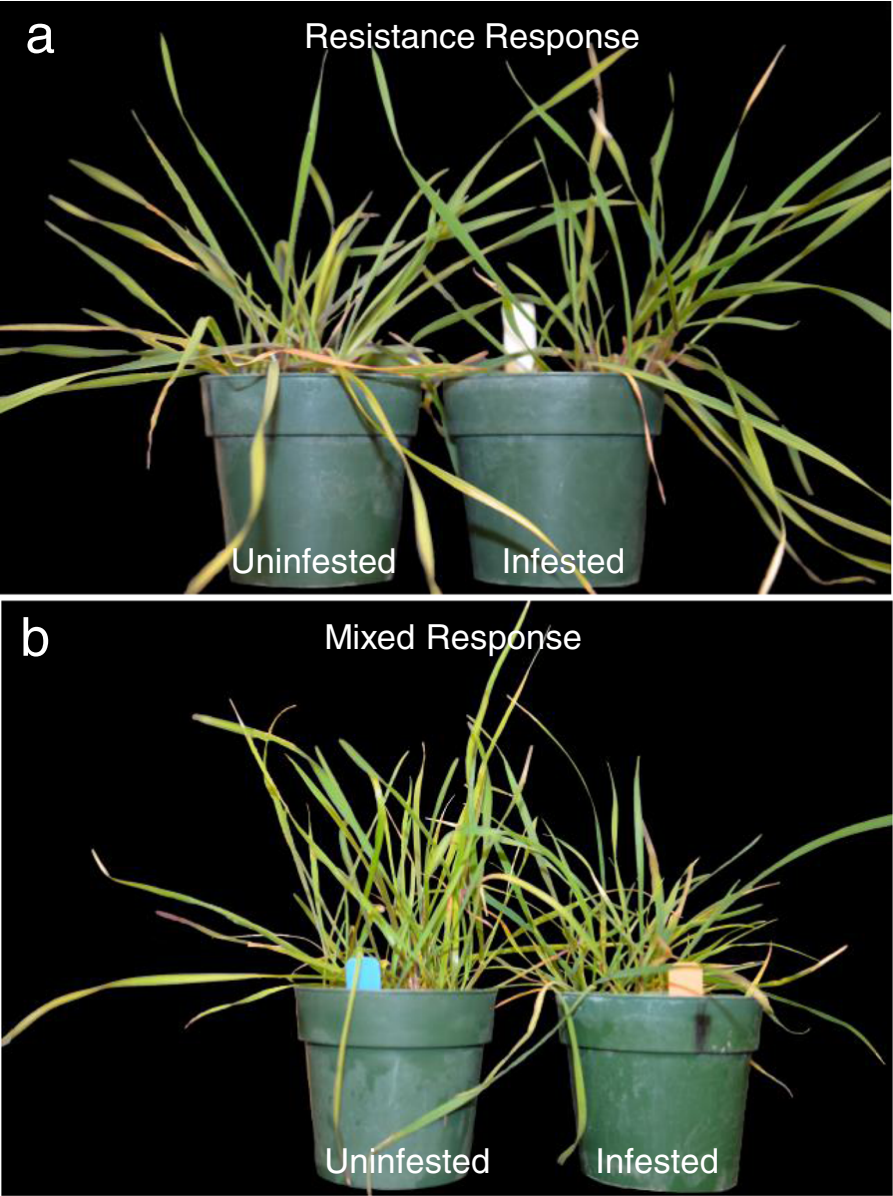
Plant growth in Hessian fly infested and uninfested *Ae. tauschii* accessions. **a** Uninfested and infested pots with TA2473 plants, representative of resistance response to larval feeding. **b** Uninfested and infested pots with TA2452 plants, representative of mixed response to larval feeding

Leaf growth was measured in *Ae. tauschii* accessions following Hessian fly infestations 17 DAH for biotype L, and 24 DAH for *vH13-*infested plants (Fig. 3). Accessions TA2473 and TA1651 exhibited a resistance response to both the Hessian fly biotypes, with stunting observed in leaf 2 and/or 3 followed by a recovery in growth of leaf 4 (Fig. 3a, b, c, d). Similar growth patterns were also observed in TA1644 showing a resistance response to biotype L (Fig. 3e). However, the mixed response plants of TA1644 showed stunting of only leaf 3 in response to feeding by *vH13* (Fig. 3f). Plants from accession TA1642, contrary to other resistance response accessions, did not show stunting of leaves 2 and 3, but did have accelerated growth of leaf 4, compared to the uninfested controls, in response to biotype L feeding (Fig. 3g). In contrast, TA1642 showed stunting of both leaves 3 and 4 in the mixed response plants infested with *vH13* (Fig. 3h). The mixed response plants from accession TA2452 showed stunting of only leaf 3 in response to feeding by biotype L (Fig. 3i), but both leaves 3 and 4 in response to *vH13* attack (Fig. 3j). Therefore, while the resistant homozygous *Ae. tauschii* plants showed leaf growth comparable to the uninfested control plants (Fig. 4a), the accessions showing mixed response (heterozygous) contained some plants that were stunted (Fig. 4b).

#### Cell wall permeability

**Fig. 5:**
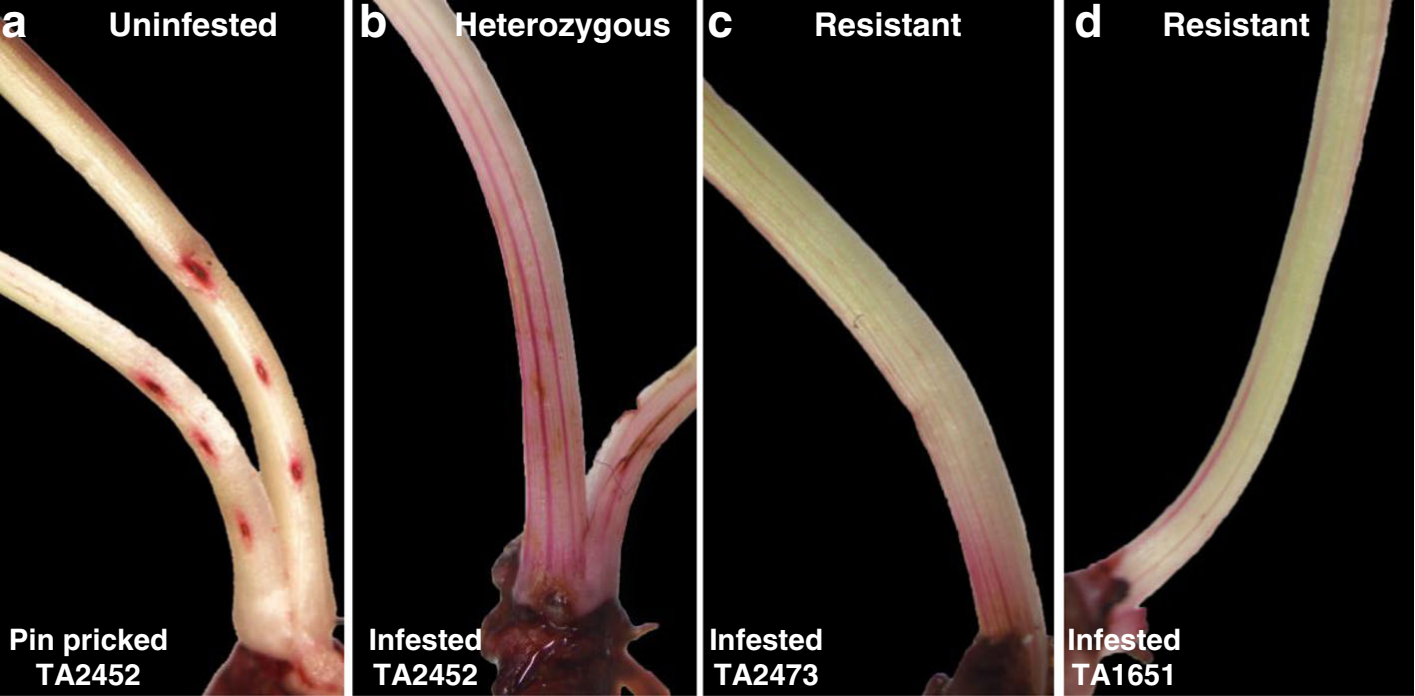
Changes in plant cell wall permeability in *Ae. tauschii* accessions. The crown, harboring the Hessian fly larvae, of plants from lines showing mixed heterozygous (TA2452) and homozygous resistant (TA2473 and TA1651) response to larval feeding were stained with neutral red (NR) to reveal intensity of cell permeability at 3 DAH. **a** Representative uninfested control TA2452 plant was pin pricked and stained to distinguish staining caused by larval feeding from that caused by physical damage; **b** NR stained TA2452 plant showing solid lines and blush around the entire length of the stem tissue; **c** NR stained TA2473 plant showing a blush restricted to the larval feeding site; **d** NR stained TA1651 plant showing solid lines restricted to the larval feeding site

To assess the cell wall permeability levels in *Ae. tauschii* accessions in response to larval feeding, biotype L-infested plants from TA2473 and TA1651 (resistance response accessions) and TA2452 (mixed response accession) were stained with neutral red (NR) and their scores compared with those obtained for resistant and susceptible hexaploid wheat lines documented previously [25]. Similar to hexaploid wheat, NR stain was absorbed only by infested *Ae. tauschii* plants but not by uninfested plants unless wounded by piercing with a minuten pin, as positive controls (Fig. 5a). Although increased permeability was observed in the resistant and mixed response *Ae. tauschii* accessions, the NR scores for heterozygous plants with live and dead larvae (TA2452) were higher on average as compared to the resistant lines (Table 2). While the NR staining appeared as blush and solid lines, spreading and covering the entire length of crown tissue in TA2452 (Fig. 5b), it was restricted to the larval feeding site at the base of the crown tissue in TA2473 (Fig. 5c) and TA1651 (Fig. 5d). The Hessian fly-resistant lines, TA2473 (Fig. 5c) and TA1651 (Fig. 5d), showed a far less intense NR staining score that resembled the hexaploid resistant wheat.

**Table 2:** Neutral Red scoring^a^ of Hessian fly-infested *Ae. tauschii* plants

Plant#	TA2452	TA2473	TA1651
1	4	3	2
2	3	2	0
3	3	3	3
4	5	1	2
5	3	4	1
6	6	na	1
Average	4.0 ± 0.5	2.6 ± 0.5	1.5 ± 0.4

^a^Plants were dissected to expose the feeding sites, stained with Neutral Red, and the intensity of red stain was scored on a scale of 0–7, according to Williams et al. [25]. Each individual plant score is shown along with the average score and standard error

*na* not available

### Molecular response of resistant *Ae. tauschii* to Hessian fly larval feeding

#### Expression profiles of Hessian fly-responsive biomarker genes

**Fig. 6:**
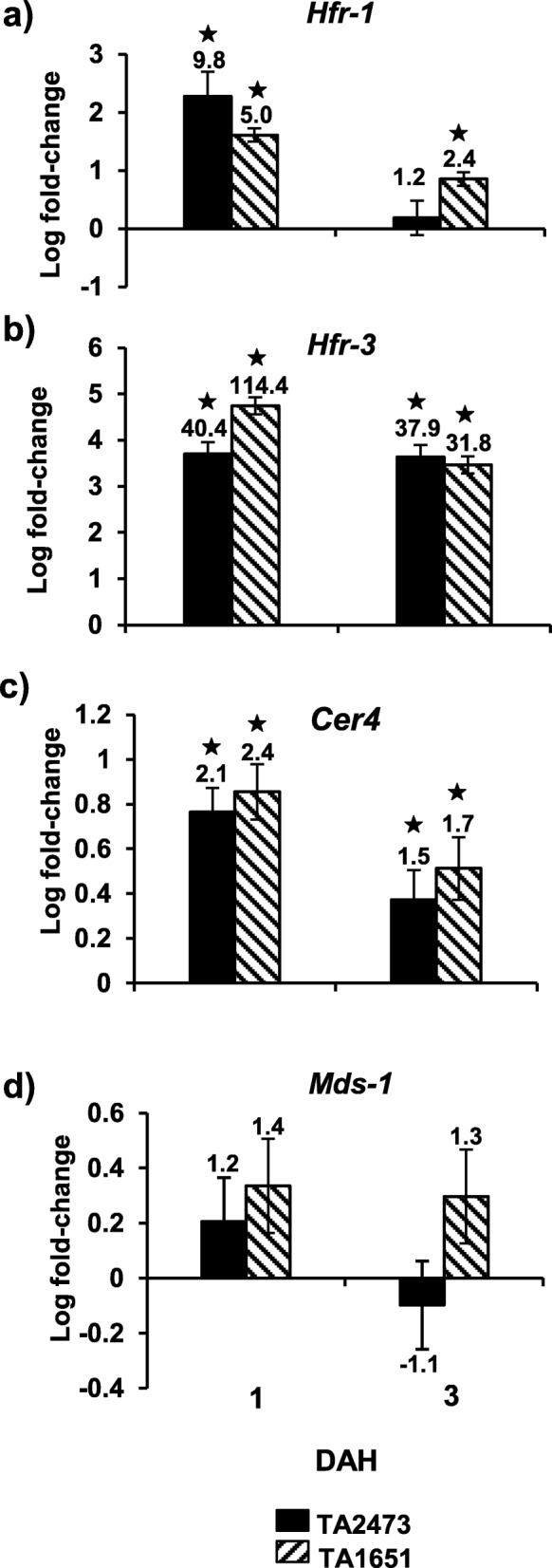
Expression of Hessian fly-responsive biomarker genes in Hessian fly-resistant *Ae. tauschii* accessions. Transcript levels of **a**) *Hfr-1* (Hessian fly response gene 1), **b**) *Hfr-3* (Hessian fly response gene 3), **c**) *Cer4* (Fatty acyl CoA reductase), and **d**) *Mds-1* (*Mayetiola destructor* susceptibility gene 1) quantified by qRT-PCR in infested and uninfested TA2473 (solid bar) and TA1651 (diagonal bar) lines at 1 and 3 DAH time-points. Values are plotted as the log fold-change of infested compared to uninfested control plants with standard error bars for 3 biological replicates. Statistically significant (*p* < 0.05) differences are indicated by ‘*’ with linear fold-change values above each bar

Transcript profiling studies were undertaken with a set of genes that serve as key biomarkers for wheat incompatible and compatible interactions. These included *Hfr-1* (Hessian fly response gene 1), *Hfr-3* (Hessian fly response gene 3), *Cer4* (Coenzyme A reductase), and *Mds-1* (*Mayetiola destructor* susceptibility 1) genes. Both *Hfr-1* and *Hfr-3* genes showed increased transcript accumulation in the two resistant TA2473 and TA1651 lines infested with biotype L compared to their uninfested controls at 1 and 3 DAH time-points (Fig. 6a, b). Transcripts of *Hfr-1* at 1 DAH were 9.8- (*p* < 0.01) and 5.0-fold (*p* < 0.001) higher in TA2473 and TA1651, respectively (Fig. 6a). Increased transcript levels of *Hfr-3*, as high as 40- to 114-fold (*p* < 0.0001) by 1 DAH, and 32- to 38-fold (*p* < 0.001) by 3 DAH, were observed in the *Ae. tauschii* accessions (Fig. 6b). Transcript levels of *Cer4* increased in TA2473 (2.1 fold, *p* < 0.001) and TA1651 (2.4 fold, *p* < 0.001) as compared to their uninfested control plants at 1 DAH (Fig. 6c). *Mds-1* did not show significant expression in either TA2473 or TA1651 (Fig. 6d).

#### Oxidative burst is involved in Ae. tauschii defense against hessian fly

**Fig. 7:**
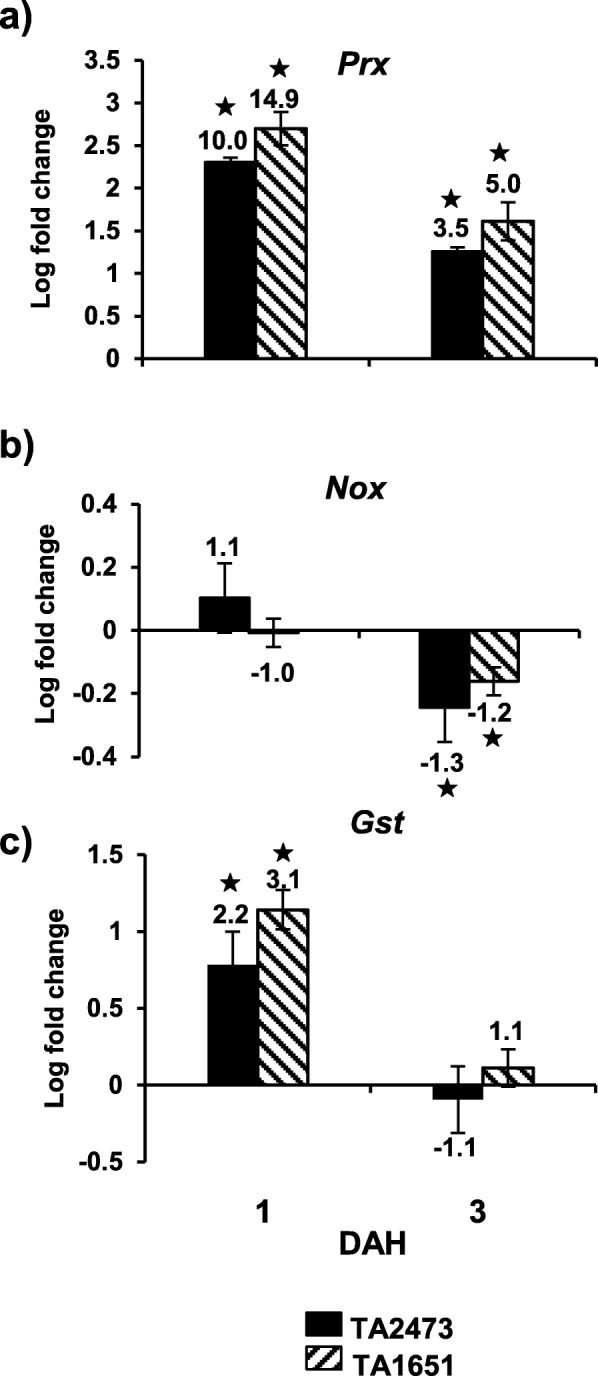
Expression of genes involved in the oxidative burst pathway in Hessian fly-resistant *Ae. tauschii* accessions. Transcript levels of **a**) *Prx* (Class III peroxidase), **b**) *Nox* (NADPH-dependent oxidase), and **c**) *Gst* (Glutathione S-transferase) quantified by qRT-PCR in infested and uninfested TA2473 (solid bar) and TA1651 (diagonal bar) wheat lines at 1 and 3 DAH time-points. Values are plotted as the log fold-change of infested compared to uninfested control plants with standard error bars for 3 biological replicates. Statistically significant (*p* < 0.05) differences are indicated by ‘*’ with linear fold-change values above each bar

To determine if reactive oxygen species (ROS) were involved in defense against Hessian fly attack in *Ae. tauschii*, despite the lack of a visible HR, we investigated the transcript profiles of genes involved in ROS production and scavenging (Fig. 7). Hessian fly-infested *Ae. tauschii* accessions showed up-regulation of the ROS-producing gene, *Prx*, encoding class III peroxidase but not of the NADPH-dependent oxidase-encoding gene, *Nox* (Fig. 7). While transcripts for *Prx* increased significantly in TA 2473 (10 fold, *p* < 0.0001) and TA1651 (14.9 fold, *p* < 0.001) as compared to their uninfested control plants (Fig. 7a), the transcripts for *Nox* were either down-regulated or not significantly expressed (Fig. 7b) by 1 and 3 DAH in the *Ae. tauschii* accessions. The mRNA levels for *Gst* gene encoding glutathione S-transferase (Fig. 7c), a ROS-scavenging enzyme also increased by 1 DAH (2.2- and 3.1-fold up-regulation).

#### Phenylpropanoids as a defense strategy in Ae. tauschii resistance

**Fig. 8:**
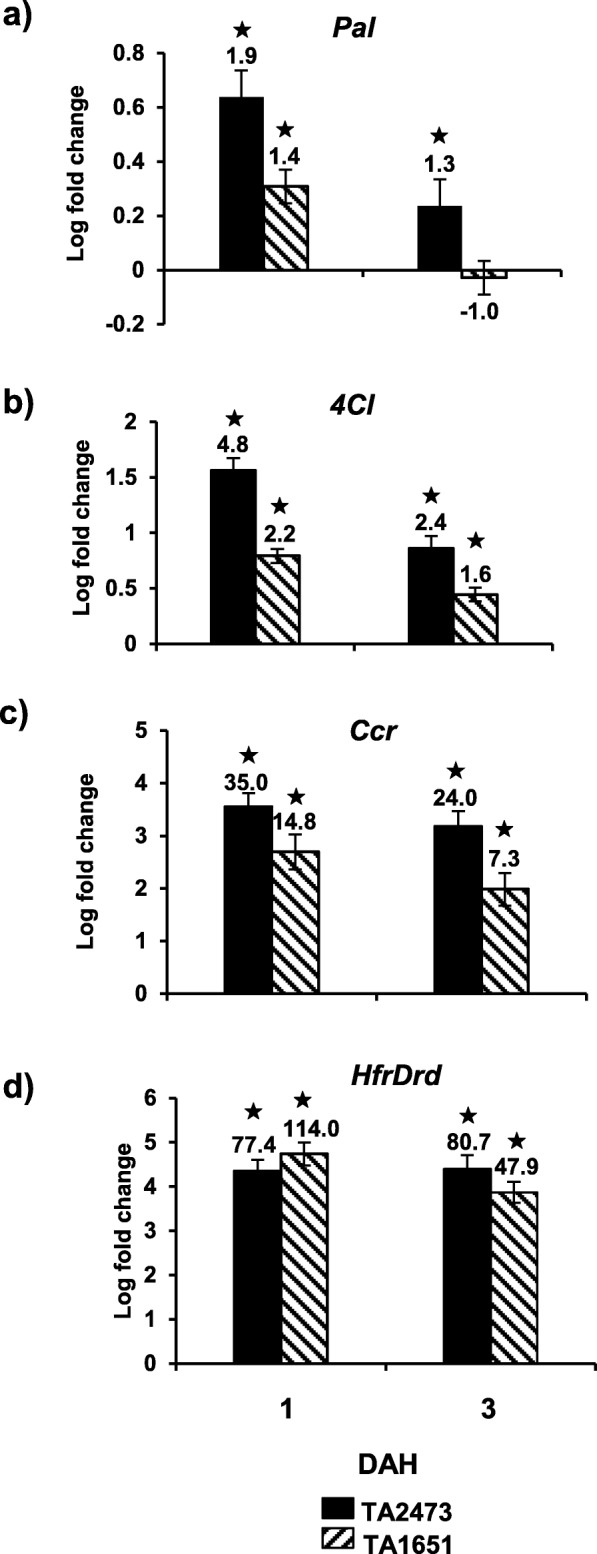
Expression of genes involved in biosynthesis of phenylpropanoids in Hessian fly-resistant *Ae. tauschii* accessions. Transcript levels of **a**) *Pal* (Phenylalanine-ammonia lyase), **b**) *4Cl* (4-coumarate-CoA ligase), **c**) *Ccr* (Cinnamoyl-CoA reductase), and **d**) *HfrDrd* (Hessian fly-responsive disease resistance dirigent-like) quantified by qRT-PCR in infested and uninfested TA2473 (solid bar) and TA1651 (diagonal bar) wheat lines at 1 and 3 DAH time-points. Values are plotted as the log fold-change of infested compared to uninfested control plants with standard error bars for 3 biological replicates. Statistically significant (*p* < 0.05) differences are indicated by ‘*’ with linear fold-change values above each bar

Transcripts for three key genes encoding PAL (phenylalanine-ammonia lyase), 4CL (4-coumarate-CoA ligase) and CCR (cinnamoyl-CoA reductase), involved in the phenylpropanoid biosynthetic pathway, were induced in both resistant *Ae. tauschii* accessions (Fig. 8). The transcripts for *Pal* and *4Cl* increased only moderately (Fig. 8a, b) as compared to transcripts of *Ccr* (Fig. 8c), that showed a much higher level of expression. The transcripts for *Ccr*, increased dramatically to 35.0- (*p* < 0.0001) and 14.8-fold *(p* < 0.00001) by 1 DAH (Fig. 8c) as compared to transcripts for *4Cl*, which increased only 4.8- and 2.2-fold (*p* < 0.01) by 1 DAH (Fig. 8b) for TA2473 and TA1651, respectively. The high levels of *Ccr* transcripts were maintained even at 3 DAH (24- and 7.3-fold; Fig. 8c). At 1 DAH, *HfrDrd* (Hessian fly-responsive disease resistance dirigent-like protein-encoding gene) transcripts increased by 77-fold in TA2473 and 114-fold in TA1651 compared to the uninfested plants. Elevated levels (81- and 48-fold in TA2473 and TA1651, respectively) of *HfrDrd* transcripts were also observed at 3 DAH (Fig. 8d).

## Discussion

The complex genome of hexaploid wheat has rendered functional genomics of candidate Hessian fly-responsive genes [26–33] challenging [34]. The use of diploid *Ae. tauschii* wheat could overcome this problem by eliminating the need to individually target all three homeologous loci (A, B and D), thereby making the process less tedious and time-consuming [23, 35]. Keeping this in view, the current work investigates the phenotypic and molecular responses of *Ae. tauschii* accessions to feeding by Hessian fly larvae. This study differs from *Ae. tauschii* screening work done previously [15] as the evaluations here were done using: (i) two different Hessian fly biotypes, L and *vH13*; (ii) additional *Ae. tauschii* accessions, TA2452 and TA2473, used in the phenotypic response evaluation experiments; and (iii) characterization of molecular responses. Identification of *Ae. tauschii* lines that exhibit responses comparable to that of hexaploid wheat could serve as potential surrogates for genetic manipulations to decipher molecular wheat-Hessian fly interactions.

The five *Ae. tauschii* accessions selected for phenotypic screening to Hessian fly biotypes are donors of various, well-documented Hessian fly resistance genes that have been introgressed into modern-day hexaploid wheat cultivars (Table 1). Screening revealed plants of TA2473 and TA1651 to be homozygous resistant where all the larvae die in the 1st-instar developmental stage resembling the incompatible (resistant) hexaploid wheat-Hessian fly interaction [14]. However, plants of TA2452 exhibited a mixed response to Hessian fly larval attack comprising of both resistant plants with all larvae dead by 7 DAH, as well as plants having both dead and live 2nd-instar larvae on the same leaf sheath. While plants for TA1644 and TA1642 were also homozygous resistant in response to biotype L attack, these accessions showed a mixed response to feeding by *vH13*. Therefore, unlike the susceptible hexaploid wheat where all larvae are in 2nd-instar stage by 7 DAH and pupate between 17 and 20 DAH [14], the heterozygous *Ae. tauschii* accessions showed presence of both dead larvae and 2nd-instar live larvae (Fig. 1b) by 7 DAH, and some biotype L and *vH13* larvae successfully pupated while others failed to pupate (Fig. 1c, d). Presence of both virulent and avirulent larvae in the mixed response, heterozygous plants of *Ae. tauschii* accessions appears to mimic some form of systemic induced susceptibility, maybe due to obviation of resistance [36]. Although occurrence of systemic induced susceptibility has been well-documented in plant-microbe interactions [37, 38] it is uncommon in plant-insect interactions [36]. It is proposed that using a highly specific and intimate relationship, a single Hessian fly larva has the ability to induce resistance or susceptibility in host plant [39]; and avirulent larvae are able to survive in the presence of virulent Hessian fly larvae [40, 41]. It is conceivable that the *Ae. tauschii* accessions showing a mixed response start out being resistant. However, due to some unknown mechanism a single larva becomes virulent and is able to breakdown resistance, in the process rescuing some of the avirulent larvae residing on the same plant. The plants exhibiting mixed response could plausibly be Hessian fly-tolerant lines, and additional studies are needed to prove the breakdown of resistance that allows some larvae to grow and pupate.

Thus, the phenotypic evaluation results revealed conclusively that four of the five *Ae. tauchii* accessions used in the current study were homozygous resistant to biotype L, and two accessions were resistant to *vH13* flies. The accessions TA1642 and TA1644 were previously shown to exhibit a homozygous resistance response to feeding by biotype D larvae [15]. Based on phenotypic screening, from the current and the previous study [15] it is amply clear that the TA1651 accession exhibits a resistance response to all three larval biotypes (L, D, and *vH13*). These newly identified resistant *Ae. tauschii* accessions could serve as potential proxies to undertake functional analyses of candidate Hessian fly-responsive/resistance genes. None of the accessions resembled a true compatible interaction (susceptible plant) comparable to hexaploid wheat cultivars, where all plants are susceptible, in response to the Hessian fly biotypes used in the current study.

Hypersensitive response (HR) is a defense reaction observed in plants at the pathogen attack site as a result of rapid production of reactive oxygen species (ROS) radicals leading to cell death, visible as necrotic lesions on the leaf surface. While some resistant wheat lines do develop HR-like lesions ([42], S. Subramanyam & J. Nemacheck unpublished data), they are not present in most resistant wheat lines [43, 44]. We evaluated the five *Ae. tauschii* accessions for their ability to produce lesions as an indication of HR to Hessian fly larval attack. Dark necrotic lesions were observed only in accessions that showed a mixed response and mostly on heterozygous plants having both live and dead larvae. The role of HR as a resistance-associated trait in plant-insect interactions, including the wheat-Hessian fly interactions, is still unclear [45, 46]. A few studies document HR as observed necrosis and cell wall collapse at sites where the larvae are found on the plants during gall midge (*Orseolia oryzae*) interactions with rice plants [47], and in response to sucking/piercing insects [48]. However, it is often difficult to determine if plant cell death is a result of disrupted feeding once the insects are killed by certain defense products or the cause for insect mortality [46]. Our results indicate that resistant *Ae. tauschii* accessions lacking HR-like lesions resemble several of the other resistant hexaploid *T. aestivum* cultivars that do not exhibit HR-like response following Hessian fly larval attack. Our results further suggest that HR-like responses in *Ae. tauschii* are not associated with resistance. Further biochemical and molecular studies will be necessary to determine if these lesions are some kind of persistent defense response to counter stress from the surviving larvae, and/or to prevent some 2nd-instar larvae from pupating and completing their life cycle.

Injury caused by Hessian fly larval feeding on susceptible hexaploid wheat cultivars manifests itself in the form of darker leaves along with stunted growth [2]. In such susceptible plants, the larvae rapidly inhibit leaf elongation with the newly formed leaf 3 being significantly shorter than the uninfested control by 3 DAH [14]. At 10 DAH, leaf 4 of susceptible plants are also very stunted and no longer elongating, even though larvae did not reside on this leaf [14]. Plausibly, resources in the susceptible wheat, by this time, are reallocated from leaf growth to development of a nutritive tissue in susceptible wheat, as reported for many other gall forming insects [49]. In contrast, although leaves on the resistant hexaploid wheat do exhibit some measure of leaf stunting, it is observed only for leaves that are actively growing while the larvae are attempting to feed. Once the larvae die by 5 DAH, as compensation for leaf stunting, the plants undergo precocious initiation, accelerated growth of upper leaves, and end up having the same leaf length as compared to the uninfested controls [14]. Leaf growth trends in plants exhibiting homozygous resistance response (TA2473, TA1651, and TA1644) resembled those observed in resistant hexaploid wheat with leaf 2 and 3 showing stunting and recovery in growth of leaf 4 (Fig. 3a, c, e). In plants from mixed response TA2452 accession only leaf 3 was stunted in response to feeding by biotype L (Fig. 3i), but both leaves 3 and 4 were stunted in response to *vH13* attack (Fig. 3j). It is possible that stress caused by larval probing is responsible for the initial stunting observed (leaves 2 and 3), in general, in the resistant plants, irrespective of the biotype used. This is followed by countering of the stress by the plant’s defenses that results in regaining leaf growth comparable to that of the uninfested controls (Fig. 4a). Compatible (susceptible) hexaploid wheat-Hessian fly interactions show a dramatic stunting as compared to resistant or uninfested plants [14]. However, although TA1642 and TA2452 contained some plants displaying stunting of the upper leaf (Fig. 4b) and pupated larvae, they do not resemble a true compatible interaction where none of the plants are resistant.

Salivary secretions from Hessian fly larvae target the cell walls in the epidermal layer of both host [25] and nonhost [13] plants, which is considered as the first line of defense against herbivory [50, 51]. Permeability studies via staining with neutral red (NR) revealed a two-way exchange of molecules during plant-Hessian fly interactions [13, 25]. Sustained increased permeability during compatible interactions indicates effective delivery of salivary effectors resulting in physiological and metabolic changes in the susceptible plant, leading to a nutritionally rich environment conducive for larval establishment [25]. Transient and limited permeability at early time-points during incompatible interactions are required for the delivery of defense toxins and proteins to the larvae, preventing them from establishing permanent feeding sites and completing their development [25]. In a wounded plant NR stain enters the cell wall and spreads mainly in the major vasculature. Resembling the hexaploid wheat, NR stain was absorbed only by infested *Ae. tauschii* plants but not by uninfested plants (Fig. 5). Although the NR scores in the mixed response accession (4.0 ± 0.5) were higher than the resistant accessions (Table 2), they were not comparable with the scores of 6 to 7 observed in susceptible hexaploid wheat [25]. The relatively increased staining in the heterozygous *Ae. tauschii* (TA2452) plants (Fig. 5b) could be due to the presence of live larvae that are attempting to make the plant tissue more permeable for increased flow and delivery of nutrients for the developing larvae. The far less intense NR staining score for Hessian fly-resistant *Ae. tauschii* accessions, TA2473 (Fig. 5c) and TA1651 (Fig. 5d) resembled the hexaploid resistant wheat suggesting that only a limited area of permeability is induced to possibly deliver host defense molecules to the larvae and prevent them from establishing permanent feeding sites [25].

Phenotypic characterization identified two accessions, TA2473 and TA1651, which exhibited a homozygous resistance response to both biotype L and *vH13* feeding (Table 1), having traits resembling the resistant hexaploid wheat documented previously. We hypothesized that resistant *Ae. tauschii* accessions would also resemble the resistant hexaploid wheat at the molecular level. To test our hypothesis, we carried out transcript profiling of Hessian fly-responsive biomarker genes. *Hfr-1* (Hessian fly response gene 1) and *Hfr-3* (Hessian fly response gene 3) are genes encoding a mannose- and chitin-binding lectin, respectively, that were chosen because these two defense response genes: (i) show increased transcript accumulation in resistant wheat within 2 DAH as compared to susceptible wheat and uninfested control plants [33, 52]; and (ii) possess antifeedant and insecticidal properties that play a significant role in plant defense [53, 54]. As expected, similar trends in up-regulation for these genes were observed in the two resistant accessions, TA2473 and TA1651, resembling the resistant hexaploid wheat. *Hfr-3,* is the most responsive gene in resistant hexaploid wheat to Hessian fly larval attack, with transcripts as high as 100-fold [52]. Similar to hexaploid resistant wheat, *Hfr-3* transcript levels were also high in the *Ae. tauschii* accessions. These results indicate the possible involvement of lectins as key components of an early defense strategy in *Ae. tauschii* lines against Hessian fly larvae, probably by disrupting the midgut microvilli and blocking nutrient absorption as observed previously in hexaploid resistant wheat [33, 53, 55]. *Cer4* encodes an alcohol-forming fatty acyl-Coenzyme A reductase and is involved in the production of protective cuticular waxes [56]. Earlier studies demonstrated an increase in *Cer4* transcripts (3-fold) during incompatible wheat-Hessian fly interactions as compared to the compatible interactions and uninfested control plants at 1 DAH [57]. Resembling the trends in resistant hexaploid wheat, transcript levels of *Cer4* also increased in the resistant *Ae. tauschii* accessions (Fig. 6c). Another key biomarker Hessian fly-responsive gene is *Mds-1* (*Mayetiola destructor* susceptibility 1) that encodes for a heat shock protein and governs wheat susceptibility to this dipteran pest [30]. *Mds-1* is not significantly expressed in resistant wheat genotypes and RNAi-mediated silencing of the gene confers immunity against several Hessian fly biotypes in susceptible wheat cultivars [30]. Similar to other resistant hexaploid wheat genotypes, *Mds-1* was not differentially expressed in *Ae. tauchii* resistant accessions. Thus, the transcript profiles of all tested Hessian fly-responsive biomarker genes indicate that molecular responses in the *Ae. tauschii* resistant accessions resemble those observed in hexaploid resistant wheat, making them an ideal model system for genetic manipulations and functional characterization of candidate defense-response and resistance genes.

A key defense strategy in plants, to counter biotic stress, is the production of ROS radicals, causing an oxidative burst and resulting in a zone of cell death (necrotic lesions) around the stress area [58]. Although visible necrotic lesions are associated with traditional HR, it is not a conclusive indication of oxidative burst at the molecular level. This is especially true with Hessian fly-resistant genotypes that show no signs of visible HR but exhibit increased transcripts of genes involved in ROS-production [44]. Another indication of oxidative burst in the Hessian fly-resistant wheat lacking HR, is the elevated transcripts of ROS-scavenging enzymes, which deplete the ROS radicals [44]. As we discussed earlier, similar to several HR-lacking Hessian fly-resistant wheat lines, resistant accessions TA2473 and TA1651 also lacked necrotic lesions in response to feeding by biotype L and *vH13* larvae (Table 1). Hessian fly-infested accessions showed increased transcripts for both ROS-producing (*Prx*) and-scavenging (*Gst*) genes (Fig. 7). *Nox*, another ROS-producing gene did not show significant expression in *Ae. tauschii* resistant plants. Our result suggests the involvement of class III peroxidase in resistance to Hessian fly instead of the classical *Nox*-mediated oxidative burst mechanism in *Ae. tauschii*. Class III peroxidases have been implicated to be one of the likely sources of elevated ROS-production, instead of NADPH-dependent oxidase, during incompatible hexaploid wheat-Hessian fly interactions [44]. Increase in mRNA levels for ROS-scavenging *Gst* gene as early as 1 DAH further corroborates the involvement of ROS in resistant *Ae. tauchii* in response to larval attack. While the role of ROS and HR in plant defense against pathogens is well-investigated [59], their putative role in plant defense against insects is still unclear [13, 43, 60–62]. It is amply clear from transcript profiling studies that there is no correlation between a physical HR (in the form of necrotic lesions) and resistance despite the presence of a strong oxidative burst in the resistant *Ae. tauschii* accessions and the increased ROS-generation could plausibly be playing a direct role in larval death.

Plant secondary metabolites such as phenylpropanoids are induced in response to insect herbivory and play an important role in plant defense [63–66]. These are produced through the shikimate pathway and their biosynthesis starts with the formation of phenylalanine that is catalyzed to coumaric acid via *Pal* and subsequently catalyzed via *4Cl* and *Ccr* to flavonols or lignins, respectively [67]. Transcripts for these three key genes encoding PAL, 4CL and CCR were induced in both resistant *Ae. tauschii* accessions (Fig. 8). The expression profiles for these genes are similar to transcript patterns observed in host hexaploid wheat and nonhost *B. distachyon* responses to Hessian fly [13, 27]. The transcripts for *Pal* and *4Cl* increased only moderately as compared to transcripts of *Ccr* that increased dramatically as early as 1 DAH and maintained at high levels even by 3 DAH in the resistant *Ae. tauschii* plants. *Ccr* is the first committed enzyme of the lignin branch biosynthetic pathway [68]. These results indicate the possible significant involvement of lignins in *Ae. tauschii* defense against Hessian fly larval attack. Lignins, a phenolic heteropolymer, defend plants from herbivory by increasing leaf toughness and decreasing leaf nutritional content, thereby hampering insect feeding and reducing fecundity [69]. Liu et al. [27] observed strong up-regulation of genes involved in lignin biosynthesis during incompatible interactions and down-regulation in the compatible interactions. Elevated abundance of *HfrDrd* transcripts, a gene encoding a dirigent-like protein, was observed in resistant *Ae. tauschii* accessions (Fig. 8d) similar to resistant hexaploid wheat [28] in response to Hessian fly larval attack. Dirigent proteins mediate free radical coupling of monolignol plant phenols to yield the cell wall polymers lignins and lignans [70, 71]. Increased *HfrDrd* mRNA mediates lignin formation leading to wall fortification and reinforcement, making the host plant cell wall a barrier against larval attack and preventing the pest from hijacking the host cellular machinery [28]. Additionally, a strong correlation has been documented between elevated transcripts of *Pal*, other phenylpropanoid biosynthesis enzymes, and peroxidases leading to increase in phenylpropanoids and lignin precursors in hypersensitive plants, and resistance to fungi [72]. Participation of class III plant peroxidases in lignin synthesis has been studied in many plant species [73]. The increased transcripts of *Prx* (Fig. 7a) may be directed towards increased lignification in the resistant *Ae. tauschii*, in addition to ROS-generation, as an added defense strategy.

## Conclusions

With recent advances in whole-genome sequencing and gene-editing tools, manipulations to express or silence target genes for functional genomics have become extremely feasible in several less complex monocots and dicots. However, modification of gene targets in modern day hexaploid wheat requires a greater degree of optimization due to the complexity of the genome [74]. In the current study we have identified Hessian fly-resistant *Ae. tauschii* accessions that share similarities to hexaploid wheat in their phenotypic and molecular responses to larval feeding. Resembling the resistant hexaploid host wheat, Hessian fly-resistant *Ae. tauschii* accessions mount an early defense strategy involving the production of antifeedant proteins (lectins), secondary metabolites and ROS radicals that potentially counter larval extra-oral salivary plant cell-degrading proteases, fortify the cell wall and prevent the Hessian fly larvae from establishing permanent feeding sites. The characterizations carried out here have amply validated the suitability of *Ae. tauschii* as an ideal tool for functional genomics of candidate Hessian fly-responsive genes that are of immense importance in crop improvement strategies.

## Methods

### Insect material

Two Hessian fly (*Mayetiola destructor*) stocks, biotype L and *vH13*, were used for infestations in the current study. Biotype L stocks were field populations collected from Posey county, Indiana, while *vH13* stocks were lab cultured. Both stocks were maintained in diapause at 4 °C at the USDA-ARS Crop Production and Pest Control Research Unit in West Lafayette, IN, following the methods described by Sosa and Gallun [75]. The purity of biotype L stock was tested by infesting wheat lines ‘Monon’, ‘Magnum’, ‘Caldwell’ and ‘Seneca’ harboring *H3*, *H5*, *H6* and *H7H8* resistance genes, respectively, resulting in compatible interactions, as expected. Purity of *vH13* stocks was assessed by infesting wheat lines ‘Iris’ (harboring *H9*) and ‘Molly’ (harboring *H13*) and, as expected, yielded incompatible and compatible interactions, respectively.

### Plant material

Five accessions of *Aegilops tauschii,* were used in the current study to evaluate for resistance to biotype L and *vH13* Hessian flies. Seeds for *Ae. tauschii* accessions TA2452 (*H13*) [76], TA1644 (*H22*) [77], and TA2473 (*H26*) [78] were obtained from the Wheat Genetics Resource Center, Kansas State University (Manhattan, KS), and seeds for TA1651 (*H32*) [7] and TA1642 (*H23*) [76] were procured from the USDA-ARS National Small Grains Collection (Aberdeen, ID).

### Plant growth and infestation

Fifteen seeds of each wheat line per pot were planted in 4-in. pots containing Pro-Line growing mix (Jolly Gardener Products Inc., Poland Spring, ME), with a layer of Fertilome time-release fertilizer (19–6-12; Voluntary Purchasing Groups Inc., Bonham, TX) and covered with Vermiculite (Perlite Vermiculite Packaging Industries, North Bloomfield, OH). The pots were watered thoroughly and placed at 4 °C for 1 week (to allow for uniform germination) and then moved to a Conviron growth chamber (Controlled Environment Ltd., Winnipeg, Manitoba, Canada) set at 18 °C with 60% humidity with a photoperiod of either 16/8 h day/night cycle for screening resistance to Hessian fly, or 24 h photoperiod for gene expression tissue collections. At the 2-leaf stage, all pots were covered with vented cups and wheat seedlings were infested with 6 female and 2 male Hessian flies per pot.

### Evaluation of Hessian fly resistance

For evaluating Hessian fly resistance in the *Ae. tauschii* accessions, 3 pots of each wheat line were infested with biotype L or with *vH13* Hessian fly stocks. One additional pot for each plant-insect interaction was left as an uninfested control. For each line 8–15 infested plants per interaction were dissected 7 days after egg hatch (DAH) and 17 (for biotype L-infested plants) or 24 (for *vH13*-infested plants) DAH, and were scored for number of dead (avirulent insect phenotype with red, dead larvae) or live larvae (virulent insect phenotype with white larvae, or larvae with green guts, or pupated larvae), presence/absence of necrotic lesions (as an indication of a potential hypersensitive response) on the leaf sheath, and stunting (susceptible plant phenotype). Larvae from representative plants for each line were placed on double-sided tape (3 M, Maplewood, MN) on a glass slide and whole leaf sheaths harboring larvae were photographed using the DP21 camera system on a SZX2 stereomicroscope (Olympus, Center Valley, PA).

### Leaf measurements

Leaf measurements (from soil level to leaf blade tips) were taken for a set of 8–15 plants (per interaction including uninfested controls) at 17 (for biotype L-infested plants) or 24 (for *vH13*-infested plants) DAH time-points. Significant differences in leaf growth between infested and uninfested plants for each wheat line were determined by analysis of variance (ANOVA) using SAS. Multiple comparisons with Tukey’s HSD test were performed to identify significant differences in the group means among treatments. Differences were considered statistically significant if the *p* value associated with the contrast was *p* < 0.05.

### Transcript profiling

For gene expression studies, 15 seeds (per pot) for accessions TA2452 and TA1651 were planted in 4-in. pots (11 pots per wheat line) as described above. Six pots for each line were infested at the 2-leaf stage with 6 female and 2 male biotype L flies, per pot. Five pots for each line were left as uninfested controls. Tissues were collected at 1 and 3 DAH time-points for both accessions. For tissue collections, the 1st leaf was gently removed. After visually confirming for presence of larvae, the bottom 1.5 cm of infested crown tissue (feeding site) for all younger leaves were collected from 10 infested plants per time-point per biological replicate. Tissue collections from 10 uninfested plants were also performed in the same manner for the corresponding time-points. Tissues were harvested from three biological replicates. Harvested tissues were immediately frozen in liquid nitrogen and stored at − 80 °C until further use.

Frozen harvested tissues were crushed to a fine powder and used for RNA isolation with TRIzol reagent (Life Technologies Corporation, Carlsbad, CA). Total RNA from each sample was quantified using a Nanodrop (NanoDrop One, ThermoFisher Scientific, Waltham, MA) and was used as the template for the first-strand cDNA synthesis (Tetro cDNA synthesis kit, Bioline, Taunton, MA). Quantitative real-time reverse transcription PCR (qRT-PCR) was performed to quantify mRNA abundance for a selected set of biomarker genes previously documented to be associated with either resistance or susceptibility of wheat to Hessian fly larval attack. Gene-specific primers for Hessian fly biomarker genes, and genes encoding enzymes involved in secondary metabolite biosynthesis and oxidative stress pathway were designed using Primer Express 3.0 software (Applied Biosystems, Foster City, CA) and are given in Table 3. The qRT-PCR was carried out on a LightCycler 480 II instrument (Roche Diagnostics Corporation, Indianapolis, IN). Each reaction volume contained 5 μl of 2X SensiFAST SYBR No-ROX (Bioline), primers at a final concentration of 0.4 μM each, and 20 ng of cDNA template in a final volume of 10 μl. PCR parameters were as follows: 95 °C for 2 min, 40 cycles of 95 °C for 5 s, 60 °C for 10s, and 72 °C for 20s. Each sample was amplified in triplicate, giving three technical replicates for each of the three biological replicates at each time-point. Amplification of single product for each target was confirmed through melt-curve analysis. Additionally, mRNA levels of a gene encoding the housekeeping enzyme ubiquitin (Table 3) were used as endogenous control to normalize cDNA levels. Relative standard curve method (User Bulletin 2: ABI PRISM 7700 Sequence) was used to quantify transcript abundance as described in Subramanyam et al. [33]. Significant differences in the logarithm-transformed values were determined by analysis of variance (ANOVA) using the PROC Mixed procedure of SAS Software version 9.4 as described in Subramanyam et al. [31]. The ANOVA model included treatments, time-points, biological replicates, and the interaction between treatments and time-points as fixed effects. Data from the three biological and three technical replicates were combined and included as a random effect in the analysis model. Orthogonal contrasts were used to evaluate differences in treatments at each time-point and differences were considered statistically significant if the *p* value associated with the contrast was *p* < 0.05. All *p* values were adjusted using Bonferroni correction. Transcript levels in infested plants were compared to levels in uninfested controls at the same time-point.

**Table 3:** qRT-PCR primers for transcript profiling in *Ae. tauschii* accessions

Gene	Forward Primer	Reverse Primer
***Ubq***^a^ (Ubiquitin)	ggtgtctccggtatcctccaa	tgctccacaccagcagaagt
Hessian fly-responsive biomarker:		
***Hfr-1*** (Hessian fly-response gene 1)	cttaagacctctgctttctctaggtga	gatggtgatgcgctctaaacg
***Hfr-3*** (Hessian fly-response gene 3)	gtccttgctgggctgatctc	tccggtcctaggccacagta
***Mds-1*** (*Mayetiola destructor* susceptibility 1)	ccaaaagcagacagcaaccccaacc	gtcggcgaaggggtcgaacac
***Cer4*** (Fatty acyl CoA reductase)	ccgattccgcattcaacttt	gacaccagggatgtggacctt
Oxidative stress pathway:		
***Prx*** (Class III peroxidase)	agggcgccttcttcgag	aggtccatgttgctcatcttgg
***Nox*** (NADPH-dependent oxidase)	atgttcggcaacttggtgact	cgtctgctctaagaagaccactttt
***Gst*** (Glutathione *S*-transferase)	gtgccggtgctgatcca	ggcgaaagcctcgtcgat
Secondary metabolite biosynthesis:		
***Pal*** (Phenylalanine-ammonia lyase)	gcgtgaagacagtggctagga	gcgtgcgttgtggagatg
***4Cl*** (4-coumarate-CoA ligase)	gcgaagcaggtggtgttctac	gggatggagctcacgaagaag
***Ccr*** (Cinnamoyl-CoA reductase)	gttgggccctctgctacaga	caccgagccgtccagatact
***HfrDrd*** (Hessian fly-responsive disease resistance dirigent-like gene)	ttgaccagtcccaccgaca	attcaaagtgttccgtaggacg

^a^Gene used as endogenous control

### Neutral red staining

To determine whether Hessian fly larvae disrupt the integrity of epidermal cell wall layer, neutral red (NR) staining of crown tissue was carried out to assess permeability at 3 DAH for 6 plants from each of the accessions TA2452, TA2473, and TA1651 as per the method described in Williams et al. [25]. The 1st leaf from Hessian fly-infested wheat seedlings was carefully peeled off to avoid wounding during the dissection process and expose the crown tissue (feeding site). Uninfested seedlings were also dissected in the same manner and poked with a 0.2 mm minuten pin prior to staining, as positive controls, to mimic wounding. Tissue samples were soaked in aqueous 0.1% (w/v) NR stain (Sigma-Aldrich, St. Louis, MO) for 10 min, and then washed thoroughly in water. Overall intensity of red staining was scored for all plants according to the scale established in Williams et al. [25] with a score of 0 indicating no stain and 7 being a completely red crown. Following staining, photomicrographs were taken for representative plants using a DP21 camera system on SZX2 stereomicroscope (Olympus).

## Data Availability

The data and materials generated or analyzed in this study are included in this published article and available from the corresponding author on reasonable request.

## Abbreviations

ANOVA: Analysis of variance
DAH: Days after egg hatch
Het: Heterozygous
Homo: Homozygous resistant
HR: Hypersensitive response
IWGSC: International Wheat Genome Sequencing Consortium
NR: Neutral red
qRT-PCR: quantitative real-time reverse transcription PCR
ROS: Reactive oxygen species
SAS: Statistical analysis system
